# Identification of Pancreatic Injury in Patients with Elevated Amylase or Lipase Level Using a Decision Tree Classifier: A Cross-Sectional Retrospective Analysis in a Level I Trauma Center

**DOI:** 10.3390/ijerph15020277

**Published:** 2018-02-06

**Authors:** Cheng-Shyuan Rau, Shao-Chun Wu, Peng-Chen Chien, Pao-Jen Kuo, Yi-Chun Chen, Hsiao-Yun Hsieh, Ching-Hua Hsieh, Hang-Tsung Liu

**Affiliations:** 1 Department of Neurosurgery, Kaohsiung Chang Gung Memorial Hospital, Kaohsiung 83301, Taiwan; ersh2127@cloud.cgmh.org.tw; 2 Department of Anesthesiology, Kaohsiung Chang Gung Memorial Hospital, Kaohsiung 83301, Taiwan; shaochunwu@gmail.com; 3 Department of Plastic Surgery, Kaohsiung Chang Gung Memorial Hospital, Kaohsiung 83301, Taiwan; venu_chien@hotmail.com (P.-C.C.); bow110470@gmail.com (P.-J.K.); libe320@yahoo.com.tw (Y.-C.C.); sylvia19870714@hotmail.com (H.-Y.H.); 4 Department of Trauma Surgery, Kaohsiung Chang Gung Memorial Hospital, Kaohsiung 83301, Taiwan

**Keywords:** decision tree, pancreatic injury, amylase, lipase, abdominal injury, shock, traumatic brain injury, mandible fracture, maxilla fracture

## Abstract

Background: In trauma patients, pancreatic injury is rare; however, if undiagnosed, it is associated with high morbidity and mortality rates. Few predictive models are available for the identification of pancreatic injury in trauma patients with elevated serum pancreatic enzymes. In this study, we aimed to construct a model for predicting pancreatic injury using a decision tree (DT) algorithm, along with data obtained from a population-based trauma registry in a Level I trauma center. Methods: A total of 991 patients with elevated serum levels of amylase (>137 U/L) or lipase (>51 U/L), including 46 patients with pancreatic injury and 865 without pancreatic injury between January 2009 and December 2016, were allocated in a ratio of 7:3 to training (*n* = 642) or test (*n* = 269) sets. Using the data on patient and injury characteristics as well as laboratory data, the DT algorithm with Classification and Regression Tree (CART) analysis was performed based on the Gini impurity index, using the rpart function in the rpart package in R. Results: Among the trauma patients with elevated amylase or lipase levels, three groups of patients were identified as having a high risk of pancreatic injury, using the DT model. These included (1) 69% of the patients with lipase level ≥306 U/L; (2) 79% of the patients with lipase level between 154 U/L and 305 U/L and shock index (SI) ≥ 0.72; and (3) 80% of the patients with lipase level <154 U/L with abdomen injury, glucose level <158 mg/dL, amylase level <90 U/L, and neutrophil percentage ≥76%; they had all sustained pancreatic injury. With all variables in the model, the DT achieved an accuracy of 97.9% (sensitivity of 91.4% and specificity of 98.3%) for the training set. In the test set, the DT achieved an accuracy of 93.3%, sensitivity of 72.7%, and specificity of 94.2%. Conclusions: We established a DT model using lipase, SI, and additional conditions (injury to the abdomen, glucose level <158 mg/dL, amylase level <90 U/L, and neutrophils ≥76%) as important nodes to predict three groups of patients with a high risk of pancreatic injury. The proposed decision-making algorithm may help in identifying pancreatic injury among trauma patients with elevated serum amylase or lipase levels.

## 1. Background

The overall incidence of pancreatic injury is relatively uncommon and has been estimated to be 1–2% of all patients with abdominal injuries. Nontheless, when it does occur, there was overall mortality rate ranged from 5% to 13% and a pancreatic morbidity of 11% [1,2,3,4]. Furthermore, trauma to the pancreatic duct can induce autodigestion of the adjacent tissue by secreted exocrine enzymes, leading to an associated risk of erosion of adjacent vascular and visceral structures [1,5]. The clinical presentation of pancreatic injury is often subtle because of the organ’s retroperitoneal location. Diagnosis that is delayed more than 24 h has been cited as the leading cause of increased morbidity, particularly in association with other blunt abdominal trauma [6,7]. Therefore, pancreatic injury should be diagnosed as early as possible to prevent serious complications and decrease the mortality that can result from delayed diagnosis.

Pancreatic enzymes may be released into the circulation as a result of damage to tissues containing high enzyme levels or by release from the gastrointestinal tract [8,9,10]. Obstruction of the bile or pancreatic duct and bowel can further lead to direct diffusion of pancreatic enzymes from the intestinal lumen into the bloodstream [11]. In consequence, serum amylase and lipase levels are often used as a diagnostic screening tool to detect pancreatic injury. However, published reports on serum levels of amylase and lipase in patients with abdominal trauma have shown mixed results. Some studies have clearly shown the importance of serum amylase and lipase levels in diagnosing pancreatic injury [6,7,12] but others have demonstrated that initial amylase and lipase measurements are not useful screening tools for detecting pancreatic injury [13,14,15,16].

The abovementioned disagreement may be attributed to the fact that, besides the pancreas, many different organs such as the tongue, esophagus, stomach, duodenum, small bowel, and liver contain amylase and lipase [17]. Amylase in different isoforms may be released from salivary glands following trauma upon this region [18]. Moreover, in a variety of shock states, clinical studies have demonstrated evidence of ischemic pancreatic inflammation with elevated levels of pancreatic enzymes [19,20]. In a study of 164 consecutive patients who presented to the emergency department (ED) with a history of blunt abdominal trauma and who had serum pancreatic enzyme assessment, 66% of patients had associated intra-abdominal injury, with 43% involving the duodenum, 15% with associated head injury, and 51% with associated extremity injury [12]. Hence, increased serum levels of amylase and lipase are not always predictive of pancreatic injury and may also reflect nonpancreatic or extrapancreatic production. An increase in serum levels of these enzymes can be caused by a broad range of conditions in patients with trauma, such as those with blunt abdominal injury [21], intracranial bleeding [22,23], critical illness in an intensive care unit [24,25], patients recovering from shock [20,26], and those undergoing maxillofacial surgery [18].

In this study, we adopted the decision tree (DT) method to explore the variables that could be used to identify individuals at risk for pancreatic injury among trauma patients with elevated amylase or lipase levels. DT is a machine learning model, which is composed of decision rules based on optimal feature cutoff values that recursively split independent variables into different groups and predict an outcome in a hierarchical manner [27,28,29]. To identify high-risk patients with pancreatic injury in clinical decision-making from among those with elevated serum levels of amylase or lipase, we aimed to construct a model to predict pancreatic injury using the DT algorithm and data obtained from a population-based trauma registry in a level I trauma center. 

## 2. Methods

### 2.1. Study Population

After approval (reference number 201701369B0) was obtained from the institutional review board of the Kaohsiung Chang Gung Memorial Hospital, a level I regional trauma center in southern Taiwan [30,31], we searched the database of the Trauma Registry System from 1 January 2009 to 31 December 2016 and reviewed medical charts for diagnostic injury codes 863.81–863.84 and 863.91–863.94 (pancreatic injury), according to the International Classification of Diseases, 9th Revision, Clinical Modification (ICD-9-CM). All patients who presented with elevated serum levels of amylase (>137 U/L) or lipase (>51 U/L) were included in the study. Of the 971 patients identified as having elevated amylase or lipase levels, 60 were excluded due to incomplete registry data or lack of laboratory blood testing data. Finally, 911 patients were included in this study. Among these, 46 patients had sustained a pancreatic injury, which had been identified either by abdominal computed tomography examination or during a laparotomy; the other 865 patients did not have pancreatic injury. 

The following data were retrieved: sex; age; co-morbidities such as diabetes mellitus (DM), hypertension (HTN), coronary artery disease (CAD), congestive heart failure, cerebral vascular accident, and end-stage renal disease; associated injuries or illness that may induce elevated amylase or lipase, including traumatic brain injury (TBI), mandible fracture, maxillary fracture, perforated peptic ulcer (PPU), ileus, torsion of ovarian cyst, mesenteric ischemia, intestinal strangulation, and mumps; vital signs, including temperature, systolic blood pressure (SBP), heart rate (HR), respiratory rate; Injury Severity Score (ISS); Glasgow coma scale (GCS) score; Abbreviated Injury Scale (AIS) score for different regions of the body; white blood cell (WBC) and red blood cell counts, percentage of neutrophils in WBCs; levels of hemoglobin, hematocrit, platelets, blood urine nitrogen (BUN), creatinine (Cr), alanine aminotransferase, aspartate aminotransferase, total bilirubin, sodium, potassium, amylase, lipase, and glucose; international normalized ratio (INR); and shock index (SI), calculated in the ED, as HR divided by SBP.

### 2.2. Decision Tree Classifier

The 911 enrolled patients were divided in a ratio of 7:3 into a training set (*n* = 642) and a test set (*n* = 269). The training set was used for predictor discovery under supervised classification and to generate a plausible model. The test set was used to assess the performance of the model generated from the training set. Classification and Regression Trees (CART) analysis [32,33] using the rpart function in the rpart package in R, based on the Gini impurity index, was used to establish the DT classification model. CART analysis was used to search for the split on each variable to partition the data into two groups: one group of mostly “1s” (people who had sustained pancreatic injury) and another group of mostly “0s” (people who did not have pancreatic injury). The CART model partitioned the data and assigned a predicted class to each subgroup. With repetition of the same process on each predictor in the model, CART identified the best overall split by iteratively testing all possible splits and creating a specified number of nodes, until the specified stopping criteria were reached or a further reduction in node impurity became impossible [34,35,36]. In this study, the method of “cost-complexity” pruning was used to generate a sequence of simpler trees. The complexity parameter (α), a measure of how much additional accuracy a split must add to the entire tree to warrant additional complexity, was set at 0.001.

### 2.3. Multivariate Logistic Regression

For comparison, the multivariate logistic regression (LR) classifier using glm function of the stats package in R was performed. A univariate LR analysis was initially performed to identify significant predictors of pancreatic injury. All significant variables derived from univariate analysis were entered into the multiple LR using stepwise elimination to identify independent risk factors for pancreatic injury. A prediction model was developed using the calculated probability value assigned to final variables based on its regression coefficient.

### 2.4. Performance of the Decision Tree Classifier

Stratified 10-fold cross-validation was used in the test set to evaluate the predictive power of the models. Briefly, patients were randomly divided into 10 folds; the number of patients with an event was approximately equal in all folds. The model was developed using nine folds and validation on the tenth. The accuracy, sensitivity, and specificity of the DT model were calculated.

### 2.5. Statistical Analysis

Statistical analyses were performed using IBM SPSS Statistics for Windows, version 20.0 (IBM Corp., Armonk, NY, USA) and R 3.3.3. (R Foundation for Statistical Computing, Vienna, Austria). The primary outcome of the study was in-hospital mortality. Two-sided Fisher’s exact or Pearson chi-square tests were used to compare categorical data, with odds ratios (ORs) and 95% confidence intervals (CIs). The normality of continuous data was examined using the Kolmogorov–Smirnov test. Mann-Whitney *U*-tests were used to analyze non-normally distributed data, which are presented as median with interquartile range (IQR, Q1–Q3). Measures of model performance regarding the area under the curve (AUC) of the receiver operator characteristic curves (ROCs) was performed using the roc & roc.test function in the pROC package in R. *p*-Values < 0.05 were defined as statistically significant.

## 3. Results

### 3.1. Characteristics and Outcomes of Patients with Elevated Amylase or Lipase Levels

As shown in Table 1, no significant differences in sex were observed between patients with and without pancreatic injury. Compared with patients who did not have pancreatic injury, those with pancreatic injury had significantly lower rates of pre-existing HTN and DM as well as associated TBI and maxillary fracture owing to trauma accident. No significant difference in the rates of associated injury or illness including maxillary fracture, PPU, torsion of ovarian cyst, and ileus were found between the two patient groups. In addition, patients with pancreatic injury had significantly higher rates of elevated AIS scores in abdominal regions, but lower AIS scores in areas of the head, face and extremities than did patients without pancreatic injury. All the continuous data did not pass the normality examination by Kolmogorov-Smirnov test. Patients with pancreatic injury were significantly older, had significantly higher GCS, neutrophils (%), amylase and lipase levels, and INR level but lower SBP, ISS, glucose, BUN, and Cr level than patients without pancreatic injury (Table 2). Among the 911 patients, there were 327 who had sustained TBI, 65 with mandible fracture, 119 with maxilla fracture, 4 with PPU, 3 with ileus, and one patient with torsion of ovarian cyst (Table 3). Among these patients, 176 and 210 of the 327 patients with TBI had a high level of amylase and lipase, respectively; 43 and 37 of the 65 patients with mandibular fracture presented with a high level of amylase and lipase, respectively; and 75 and 70 of the 119 patients with maxillary fracture had a high level of amylase and lipase, respectively. Notably, among patients with TBI, mean levels of amylase and lipase were 172 U/L and 71 U/L, respectively. Though, maximal levels of amylase and lipase in these patients reached 1960 U/L and 767 U/L, respectively. Further, in patients with mandible or maxilla fracture, the mean amylase level was extremely high, up to around 2000 U/L; After all, the mean lipase level was around 76 U/L, albeit in some patients the lipase level reached about 542 U/L. In this study, no patients had mesenteric ischemia, intestinal strangulation, or mumps. 

### 3.2. Classification by Decision Tree Algorithm

**Figure 1:**
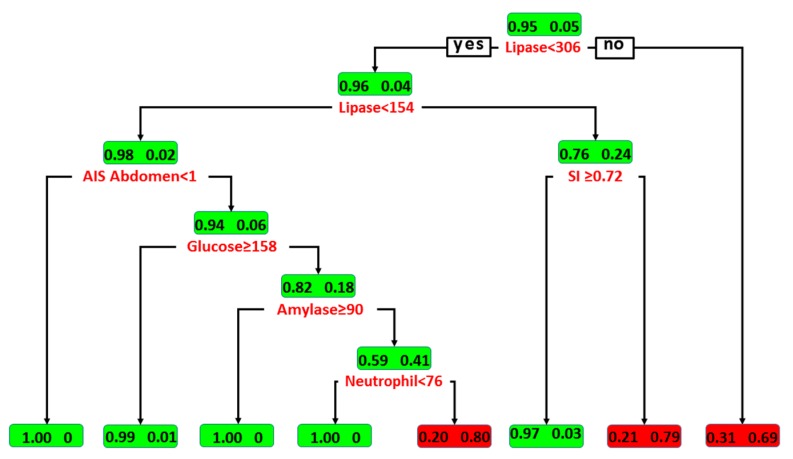
Illustration of DT model for predicting pancreatic injury in patients with elevated serum amylase or lipase levels. Boxes denote the percentage of patients with discriminating variables from CART analysis. Patients with and without pancreatic injury are indicated by the fractional number inside the right and left sides of the boxes, respectively.

**Figure 2:**
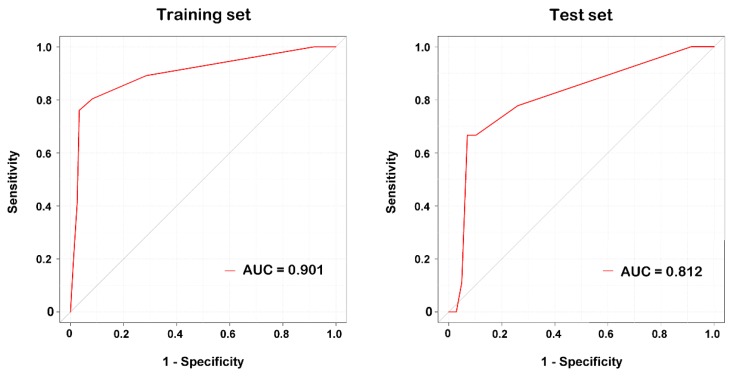
Illustration of ROC curves for the DT model in the training and test set.

As shown in Figure 1, the lipase level was identified in the DT model as the variable of the initial split, with an optimal cutoff value of <306 U/L. Among patients with lipase level ≥306 U/L, 69% had pancreatic injury and 31% did not. Among patients with lipase level between 154 U/L and 305 U/L, SI was identified as the variable of the second split, with an optimal cutoff value ≥0.72. For the node, only 3% of patients with SI ≥ 0.72 had sustained pancreatic injury. In contrast, 79% of patients with SI < 0.72 had pancreatic injury. Among patients with lipase level <154 U/L, the presence of abdominal injury (AIS abdomen < 1), glucose level ≥158 mg/dL, amylase ≥90 U/L, and neutrophils <76% served as additional predictors for the determination of associated pancreatic injury in patients. This indicated that among patients with a lipase level <154 U/L, 80% of those with abdomen injury, glucose level <158 mg/dL, amylase level <90 U/L, and neutrophil percentage ≥76% had sustained pancreatic injury. Among patients with a lipase level <154 U/L, only 1% of those with abdomen injury and glucose level ≥158 mg/dL had sustained pancreatic injury. According to classification by the DT, three groups of trauma patients with a high risk of pancreatic injury were identified (Figure 1). With all variables in the model, the DT achieved an accuracy of 97.9% (sensitivity of 91.4% and specificity of 98.3%) for the training set. In the test set, the DT achieved an accuracy of 93.3%, sensitivity of 72.7%, and specificity of 94.2%. The DT model had an AUC of 0.901 of all samples and AUC of 0.812 of test set in predicting a pancreatic injury (Figure 2).

### 3.3. Classification by Multivariate LR

The final multivariate regression models revealed that the pancreatic injury were associated with 12 independent risk factors, which included WBC, Cr, AST, ALT, Na, lipase, AIS of face, AIS of thorax, AIS of abdomen, AIS of extremity, DM (Table 4). With all variables in the model, the logistic model achieved an accuracy of 96.9% (sensitivity of 56.8% and specificity of 99.3%) for the training set. In the test set, the logistic model achieved an accuracy of 98.1%, sensitivity of 77.7%, and specificity of 98.8%.

## 4. Discussion

In this DT model, the lipase level was the first node in predicting pancreatic injury among patients with elevated pancreatic enzymes. According to well-accepted consensus, serum lipase level is the most important variable related to pancreatic injury. Elevated serum lipase level served as a more specific marker than amylase for pancreatic injury [12]. In contrast, elevated levels of pancreatic enzymes among critically injured patients in the absence of pancreatic injury are generally owing to craniofacial injuries [23,37], such that the reliability of serum amylase levels to predict pancreatic injury is questionable [23]. Furthermore, amylase level is significantly associated with the time of measurement, particularly among patients in whom amylase was measured 2 h or less post-injury [38]. However, such an association is not significant for lipase measurements [38]. The risk of patients having a pancreatic injury can be determined according to the lipase level as (1) ≥306 U/L; (2) between 154 U/L and 305 U/L; or (3) <154 U/L. Among trauma patients with elevated amylase or lipase levels, we identified three groups of patients as having a high risk of pancreatic injury using the DT model. These groups were as follows: (1) 69% of patients with lipase level ≥306 U/L had sustained pancreatic injury; (2) 79% of patients with lipase level between 154 U/L and 305 U/L and SI ≥ 0.72 had sustained pancreatic injury; and (3) 80% of patients with lipase level <154 U/L and who had sustained abdomen injury, glucose level < 158 mg/dL, amylase level <90 U/L, and neutrophils ≥ 76% had pancreatic injury. 

In this study, the maximum levels of lipase reached 767 U/L and 542 U/L in patients with TBI as well as injury to the mandible and maxilla, respectively. Thus, determination of pancreatic injury made by relying solely on the lipase level may result in a false positive diagnosis. For example, 31% of patients with lipase level ≥306 U/L in this study did not have pancreatic injury. The existence of TBI or fracture of the mandible or maxilla could explain a high serum level of lipase, as no patients had concurrent mandibular or maxillary fracture with pancreatic injury in this study; however, using trauma to the craniofacial area to exclude possible pancreatic injury among patients with high lipase levels may not be justified, particularly considering the possible occurrence of injury to the abdomen and craniofacial area; such patients may have levels of consciousness that are insufficiently alert to describe their abdominal injury.

The second important node in the prediction of pancreatic injury in this DT model was the SI. In this study, among patients with lipase level between 154 U/L and 305 U/L, an SI ≥ 0.72 determined pancreatic injury in 79% of patients. In contrast, only 3% of patients with SI ≥ 0.72 had a pancreatic injury. Histological and clinical evidence have demonstrated ischemic pancreatic inflammation in a variety of shock states [19]. Patients with an elevated level of pancreatic enzymes are at greater risk of presenting with shock [20]. The SI, which is the ratio of HR to SBP, has been used to identify hypovolemic shock in patients with trauma since 1967 [39]. SI is an easily obtained indicator of hemodynamic instability [40,41,42] and a clinical indicator of hypovolemic shock upon arrival to the ED [43]. In a study among healthy participants with blood loss of 450 ml, the SI was substantially increased whereas the HR and SBP remained within the normal ranges [44]. Classification of patients by an SI > 0.7 can preferentially select patients with adverse short-term outcomes from among those with upper gastrointestinal bleeding [45]. With SI above 0.9, the risk for trauma patients requiring massive transfusion rises substantially [46]. Takahashi et al. reported that 80% of patients with traumatic shock but without pancreatic injury had elevated serum amylase levels and 94% of them had elevated amylase owing to secretion from salivary glands [37]. This may also reflect that in the DT model of this study, SI was suitable for determining the risk of pancreatic injury according to level of lipase but not that of amylase.

In some medical centers, normal serum levels of amylase and lipase are set at 95 U/L and 38 U/L, respectively [21]. In the present study, serum levels >137 U/L and >51 U/L for amylase and lipase, respectively, were set as abnormal levels according to hospital guidelines for the Taiwanese population. The definition of high amylase and lipase levels may vary among different studies and medical institutions. In a prospective study, Mahajan et al. reported that using cutoffs of 250 U/L and 100 U/L for serum amylase and lipase levels, respectively, a combined serum amylase and lipase assessment showed 100% specificity and 85% sensitivity for predicting pancreatic injury in patients with blunt abdominal trauma [12]. In addition, Nadler et al. suggested that values of serum amylase >200 U/L and lipase >1800 U/L were useful cutoffs for detecting patients who were more likely to have major pancreatic duct injuries [47]. Obviously, the choice of cutoff value for amylase or lipase as an abnormal expression would have a remarkable impact on diagnostic accuracy. One advantage of the DT algorithm is its construction does not require any domain knowledge or parameter setting, thus making it appropriate for exploratory knowledge discovery. Unlike conventional statistical analyses that tend to identify the different variables among the compared groups, the DT algorithm uses a procedure for classifying data based on their attributes of the evaluated outcome. The nodes and their cutoff values were automatically identified as important determinative variables in the prediction of pancreatic injury. For example, despite a significantly younger age, higher lipase level, lower glucose level, and higher percentage of neutrophils among patients with pancreatic injury than those without, in this study there was even no significant difference in SI between patients with and without pancreatic injury.

Further, among patients with lipase level <154 U/L, an AIS score indicating abdominal injury is also important in determining the risk of pancreatic injury. No pancreatic injury was found in patients with lipase level <154 U/L and without an elevated AIS score for the abdomen. If there was associated injury to the abdomen indicated by the AIS score, then the levels of glucose and amylase and the neutrophil percentage provided additional information for determining the risk of pancreatic injury. According to this DT model, it is impossible to determine the occurrence of pancreatic injury with very high confidence among patients with elevated pancreatic enzymes in the serum. Anyhow, considering that few data are available for identifying these high-risk individuals to guide physicians in determining which patients will likely need further investigation, this model is easy and cost effective and may be helpful in identifying patients with a high risk of pancreatic injury. Nevertheless, the above limitation indicates that there is room for improvement in the established DT model.

Because the higher rate of patients without pancreatic injury than those with pancreatic injury would be accompanied by a high accuracy and specificity in the prediction of the illness, in that event, we would rather focus on the sensitivity between DT and multivariate LR models. In this study, the DT model had a higher sensitivity than LR in the training set (91.4% vs. 56.8%, respectively) and a comparable sensitivity than LR in the test set (72.7% vs. 77.7%, respectively). Nontheless, the present study has some additional limitations. The first of these is the selection bias associated with the retrospective study design. In such circumstance, an overfit in analyzing the collected data may be encountered and the relative small sample size would limit the value of validating the model. A prospective cohort study would get more valuable information to test the established DT model. Second, the diagnosis value of the presence of pancreatic enzymes, especially amylase level, is time dependent. Some authors have proposed that serum and lipase levels measured within 3 h after trauma have no diagnostic value [12]. Although amylase and lipase levels were checked after consultation with a general surgeon for most of our patients, measurements were performed within 3 h after trauma in some patients, which may have caused a selection bias. Third, it has been reported that higher grade injuries to the pancreas tend to result in significantly elevated mean levels of serum amylase and lipase than lower grade injuries [12]. The degree of injury to the pancreas parenchyma was not investigated in this study; thus the value of the information provided is limited. Fourth, amylase and lipase are not routinely measured in the emergency department, only for suspicion of a pancreatic injury or under the recommendation of a general surgeon; therefore, the inclusion of patients with elevated pancreatic enzymes but not those with abdominal injury in the study population may result in selection bias. Further, future studies may consider the possibility of incorporating a patient into the model which considers the patient as the most important member of the health team [48]. Finally, the study was limited to a single center; patient injury characteristics may vary from those observed at other institutions, thereby limiting the generalizability of the findings. The work carried out in multiple centers may provide more valuable information to the establishment of the DT model.

## 5. Conclusions

We established a DT model using lipase level, SI, and additional conditions (elevated AIS score indicating abdominal injury, glucose level <158 mg/dL, amylase level <90 U/L, and neutrophils ≥76%) as important nodes to predict three groups of patients with a high risk of pancreatic injury. The proposed decision-making algorithm may help to identify a pancreatic injury in trauma patients with elevated serum amylase or lipase levels.

## Figures and Tables

**Table 1:** Comparison of categorical variables of patient and injury characteristics, used to establish the decision tree model, between patients with and without pancreatic injury.

Variables		Total (*n* = 911)	Pancreatic Injury		*p*-Value
			No (*n* = 865)	Yes (*n* = 46)	
Sex	Female	325 (35.7%)	303 (35%)	22 (48%)	0.084
	Male	586 (64.3%)	562 (65%)	24 (52%)	
CVA	No	896 (98.4%)	850 (98.3%)	46 (100%)	>0.999
	Yes	15 (1.6%)	15 (1.7%)	0 (0%)	
CAD	No	885 (97.1%)	839 (96.9%)	46 (100%)	0.637
	Yes	26 (2.9%)	26 (3.1%)	0 (0%)	
HTN	No	729 (80.0%)	685 (79.2%)	44 (47.8%)	0.004
	Yes	182 (20%)	180 (20.8%)	2 (2.2%)	
CHF	No	905 (99.3%)	859 (99.3%)	46 (100%)	>0.999
	Yes	6 (0.7%)	6 (0.7%)	0 (0%)	
ESRD	No	880 (96.6%)	834 (96.4%)	46 (100%)	0.398
	Yes	31 (3.4%)	31 (3.6%)	0 (0%)	
DM	No	793 (87%)	747 (86.4%)	46 (100%)	0.003
	Yes	118 (13%)	118 (13.6%)	0 (0%)	
TBI	No	584 (64.1%)	544 (62.9%)	40 (87%)	0.001
	Yes	327 (35.9%)	321 (37.1%)	6 (13%)	
Mandible fracture	No	846 (92.9%)	800 (92.5%)	46 (100%)	0.069
	Yes	65 (7.1%)	65 (7.5%)	0 (0%)	
Maxilla fracture	No	792 (86.9%)	746 (86.2%)	46 (100%)	0.003
	Yes	119 (13.1%)	119 (13.8%)	0 (0%)	
PPU	No	907 (99.6%)	861 (99.5%)	46 (100%)	>0.999
	Yes	4 (0.4%)	4 (0.5%)	0 (0%)	
Ileus	No	908 (99.7%)	862 (99.7%)	46 (100%)	>0.999
	Yes	3 (0.3%)	3 (0.3%)	0 (0%)	
Torsion of ovarian cyst	No	910 (99.9%)	864 (99.9%)	46 (100%)	>0.999
	Yes	1 (0.1%)	1 (0.1%)	0 (0%)	
AIS (Head)	0	382 (41.9%)	346 (40%)	36 (78.3%)	<0.001
	1	114 (12.5%)	110 (12.7%)	4 (8.7%)	
	2	49 (5.4%)	47 (5.4%)	2 (4.3%)	
	3	159 (17.5%)	155 (17.9%)	4 (8.7%)	
	4	140 (15.4%)	140 (16.2%)	0 (0%)	
	5	60 (6.6%)	60 (6.9%)	0 (0%)	
	6	7 (0.8%)	7 (0.8%)	0 (0%)	
AIS (Face)	0	655 (71.9%)	611 (70.6%)	44 (95.7%)	0.002
	1	62 (6.8%)	60 (6.9%)	2 (4.3%)	
	2	182 (20%)	182 (21%)	0 (0%)	
	3	12 (1.3%)	12 (1.4%)	0 (0%)	
AIS (Thorax)	0	555 (60.9%)	515 (59.5%)	40 (87%)	0.009
	1	37 (4.1%)	35 (4%)	2 (4.3%)	
	2	71 (7.8%)	71 (8.2%)	0 (0%)	
	3	146 (16%)	144 (16.6%)	2 (4.3%)	
	4	93 (10.2%)	91 (10.5%)	2 (4.3%)	
	5	9 (1%)	9 (1%)	0 (0%)	
AIS (Abdomen)	0	547 (60%)	545 (63%)	2 (4.3%)	<0.001
	1	23 (2.5%)	23 (2.7%)	0 (0%)	
	2	165 (18.1%)	149 (17.2%)	16 (34.8%)	
	3	101 (11.1%)	89 (10.3%)	12 (26.1%)	
	4	60 (6.6%)	46 (5.3%)	14 (30.4%)	
	5	15 (1.6%)	13 (1.5%)	2 (4.3%)	
AIS (Extremity)	0	413 (45.3%)	381 (44%)	32 (69.6%)	0.008
	1	55 (6%)	53 (6.1%)	2 (4.3%)	
	2	235 (25.8%)	229 (26.5%)	6 (13%)	
	3	190 (20.9%)	186 (21.5%)	4 (8.7%)	
	4	14 (1.5%)	12 (1.4%)	2 (4.3%)	
	5	4 (0.4%)	4 (0.5%)	0 (0%)	
AIS (External)	0	770 (84.5%)	732 (84.6%)	38 (82.6%)	0.974
	1	126 (13.8%)	118 (13.6%)	8 (17.4%)	
	2	6 (0.7%)	6 (0.7%)	0 (0%)	
	3	4 (0.4%)	4 (0.5%)	0 (0%)	
	4	1 (0.1%)	1 (0.1%)	0 (0%)	
	5	1 (0.1%)	1 (0.1%)	0 (0%)	
	6	3 (0.3%)	3 (0.3%)	0 (0%)	

AIS = abbreviated injury scale; CAD = coronary artery disease; CHF = congestive heart failure; CVA = cerebral vascular accident; DM= diabetes mellitus; ESRD = end-stage renal disease; HTN = hypertension; PPU = perforated peptic ulcer; TBI = traumatic brain injury.

**Table 2:** Comparison of continuous variables of patient and injury characteristics, used in the decision tree model, between patients with and without pancreatic injury.

Variables	Total (*n* = 911) Median (IQR)	Pancreatic Injury		*p*-Value
		No (*n* = 865) Median (IQR)	Yes (*n* = 46) Median (IQR)	
Age (years)	45 (26–61)	46 (26–61)	39 (18–59)	0.039
SBP (mmHg)	133 (108–155)	133 (108–156)	121 (105–144)	0.032
RR (times/min)	20 (18–20)	20 (18–20)	20 (18–20)	0.714
Temperature (°C)	36.4 (36–36.9)	36.4 (36–36.9)	36.4 (36.1–36.8)	0.908
GCS	15 (9–15)	15 (9–15)	15 (14–15)	0.001
ISS	16 (9–24)	16 (9–24)	10 (8–18)	0.022
RBC (10^6^/µL)	4.3 (3.8–4.7)	4.3 (3.8–4.7)	4.0 (3.6–4.6)	0.115
WBC (10^3^/µL)	13.5 (9.5–18.8)	13.5 (9.5–19)	12.6 (9–14.8)	0.092
Neutrophil (%)	77.1 (65–85.8)	76.2 (64.4–85.5)	85.2 (78.8–89)	<0.001
Hb (g/dL)	12.7 (11.2–14.3)	12.7 (11.2–14.3)	12.3 (10.8–14.5)	0.300
Hct (%)	37.9 (33.6–41.9)	38.0 (33.7–42)	37.2 (32.7–41.2)	0.318
Platelets (10^3^/µL)	206 (161–251)	206 (161–253)	202 (164–230)	0.734
Glucose (mg/dL)	177 (136–179)	177 (136–179)	150 (149–168)	0.023
Na (mEq/L)	139 (137–141)	139 (137–141)	140 (137–141)	0.213
K (mEq/L)	3.6 (3.2–4.0)	3.6 (3.2–4.0)	3.7 (3.5–3.9)	0.121
BUN (mg/dL)	14 (10–18)	14 (10–18)	12.3 (8–14)	0.007
Cr (mg/dL)	0.9 (0.8–1.2)	1.0 (0.8–1.2)	0.8 (0.7–0.9)	<0.001
AST (U/L)	86 (41–184)	83 (41–189.5)	100 (61–179)	0.179
ALT (U/L)	49 (26–120)	49 (26–121)	65 (40–96)	0.190
Total bilirubin (mg/dL)	0.9 (0.7–0.9)	0.9 (0.6–0.9)	0.9 (0.8–1.0)	0.090
Amylase (U/L)	136 (84–183)	136 (84–179)	148 (87–554)	0.005
Lipase (U/L)	61 (45–89)	60 (43–84)	176 (97–375)	<0.001
INR	1.1 (1.0–1.2)	1.1 (1.0–1.1)	1.1 (1.0–1.2)	0.044
SI	0.7 (0.6–0.9)	0.7 (0.6–1.0)	0.7 (0.6–0.9)	0.589

ALT = alanine aminotransferase; AST = Aspartate transaminase; BUN = blood urea nitrogen; Cr = creatinine; GCS = Glasgow coma scale; Hb = hemoglobin; Hct = hematocrit; INR = international normalized ratio; K = potassium; Na = sodium; ISS = injury severity score; RBC = red blood cells; RR = respiratory rate; SBP = systolic blood pressure; SI = shock index; WBC = white blood cells. These continuous data was expressed with median and interquartile range.

**Table 3:** Serum amylase and lipase levels of patients with an associated injury or illness.

Variables	Total (*n*)	Amylase				Lipase			
		Median (IQR)	Max	Min	≥138 U/L (*n*)	Median (IQR)	Max	Min	≥52 U/L (*n*)
Traumatic brain injury	327	142 (87–182)	1960	12	176	56 (38–73)	767	12	210
Mandible fracture	65	160 (107–239)	843	47	43	55 (28–78)	542	2.1	37
Maxilla fracture	119	155 (105–227)	956	40	75	54 (33–75)	542	15	70
Perforated peptic ulcer	4	202 (130–254)	267	55	3	75 (49–93)	93	27	3
Torsion of ovarian cyst	1	-	84	84	0	-	72	72	1
Ileus	3	103 (89–118)	132	74	0	89 (81–132)	174	73	3

**Table 4:** Independent risk factors for pancreatic injury in multivariate logistic regression.

Independent Variables	Coefficient	Independent Variables	Coefficient
WBC	0.6911	lipase	−0.2176
neutrophil	0.1522	AIS of face	−0.3066
Cr	0.1061	AIS of thorax	−0.5152
AST	0.0094	AIS of abdomen	−1.848
ALT	0.0073	AIS of extremity	−2.1096
Na	−0.0164	DM	−19.4426
Intercept	−47.4648		
